# Anti-Inflammatory Functions of Methanol Extract from *Malus baccata* (L.) Borkh. Leaves and Shoots by Targeting the NF-κB Pathway

**DOI:** 10.3390/plants11050646

**Published:** 2022-02-26

**Authors:** Chaoran Song, Hongxi Chen, Soo Ah Kim, Jong Sub Lee, Eui Su Choung, Zhiyun Zhang, Soo-Yong Kim, Jong-Hoon Kim, Jae Youl Cho

**Affiliations:** 1 Department of Integrative Biotechnology, Biomedical Institute for Convergence at SKKU (BICS), Sungkyunkwan University, Suwon 16419, Korea; songchaoran115@163.com (C.S.); chenhx19@gmail.com (H.C.); 2 DanjoungBio Co., Ltd., Wonju 26303, Korea; sa.kim@danjoungbio.com (S.A.K.); js.lee@danjoungbio.com (J.S.L.); esavella@hanmail.net (E.S.C.); 3 State Key Laboratory of Systematic and Evolutionary Botany, Institute of Botany, The Chinese Academy of Sciences, Beijing 100093, China; zhangzy@ibcas.ac.cn; 4 International Biological Material Research Center, Korea Research Institute of Bioscience and Biotechnology, Daejeon 34141, Korea; soodole@kribb.re.kr; 5 College of Veterinary Medicine, Chonbuk National University, Iksan 54596, Korea

**Keywords:** *Malus baccata* (L.) Borkh., anti-inflammatory, NF-κB

## Abstract

*Malus baccata* (L.) Borkh. is a widely used medical plant in Asia. Since the anti-inflammatory mechanism of this plant is not fully understood, the aim of this study was to explore the anti-inflammatory function and mechanism of *Malus baccata* (L.) Borkh. methanol extract (Mb-ME). For in vitro experiments, nitric oxide production assay, PCR, overexpression strategy, immunoblotting, luciferase reporter assay, and immunoprecipitation were employed to explore the molecular mechanism and the target proteins of Mb-ME. For in vivo experiments, an HCl/EtOH-induced gastritis mouse model was used to confirm the anti-inflammatory function. Mb-ME showed a strong ability to inhibit the production of nitric oxide and the expression of inflammatory genes. Mb-ME decreased NF-κB luciferase activity mediated by MyD88 and TRIF. Moreover, Mb-ME blocked the activation of Src, Syk, p85, Akt, p50, p60, IKKα/β, and IκBα in LPS-induced RAW264.7 cells. Overexpression and immunoprecipitation analyses suggested Syk and Src as the target enzymes of Mb-ME. In vitro results showed that Mb-ME could alleviate gastritis and relieve the protein expression of p-Src, p-Syk, and COX-2, as well as the gene expression of COX-2 and TNF-α. In summary, this study implied that Mb-ME performs an anti-inflammatory role by suppressing Syk and Src in the NF-κB signaling pathway, both in vivo and in vitro.

## 1. Introduction

Inflammation is a crucial immune response that protects our bodies from diverse pathogens. When microorganisms such as Gram-negative bacteria with lipopolysaccharide (LPS) invade a human body, an innate immune response will be initiated within a few hours. Upon injury and infection, immune cells use pattern recognition receptors (PRRs) to sense exogenous infectious ligands whose molecular structures are broadly shared by pathogens and endogenous molecules that are released from dying and damaged cells [1]. Toll-like receptors (TLRs), well-characterized members of PRRs, are a membrane-bound protein [2,3]. Possessing the Toll-interleukin receptor (TIR) domain, four adaptor proteins are able to be recruited by TLRs: MyD88, TRIF, TIRAP, and TRAM [4,5]. The interactions between adaptor proteins and TLRs trigger transforming growth factor beta-kinase 1 (TAK1), leading to the activation of IKK complex-NF-κB and mitogen-activated protein kinases [6]. Consequently, extracellular signals are transduced through transcription factors, including NF-κB, JAK-STAT, and AP-1 [7,8]. Subsequently, the translocation of transcription factors is elicited. Ultimately, these processes lead to the elevated release of inflammatory cytokines; chemokines; and IFNs, such as iNOS, interleukins, and TNF-α, to protect the host from microbial infection [9,10,11,12]. Although inflammation is a vital process, uncontrolled and excessive inflammatory responses contribute to serious illnesses, such as diabetes, cancer, rheumatoid arthritis, and Alzheimer’s disease [12,13,14]. Therefore, there is an urgent demand to develop highly effective and safe anti-inflammatory drugs.

*Malus baccata* (L.) Borkh. is a common plant mainly found in China, Russia, and Korea. It has a small edible fruit with traditional medicinal properties. Extracts of *Malus baccata* exhibit considerable amounts of bioactive components, such as flavonoids; anthocyanins of a-sitosterol and ursolic acid; and fatty acid molecules of palmitic acid, ethyl palmitate, and linolein [15,16,17]. The ethanol extract from the leaves of *Malus baccata* has been reported to contain inhibitors of fatty acid synthase (FAS), which can reduce body weight in vitro [18]. In addition, phenolic ingredients in the fruits of *Malus baccata* could significantly enhance immunomodulation activity and protect spleen cells from radiation-caused damage by regulating apoptosis [19]. Juices prepared from *Malus baccata* showed a strong inhibitory effect on proliferation in HL-60 human leukemia cells and strong DPPH radical scavenging activity [20]. There is little evidence that methanol extract from *Malus baccata* (Mb-ME) has the potential to inhibit excessive inflammation responses, necessitating the present study. In addition, the anti-inflammatory function and mechanism of this plant have not been fully elucidated yet. Therefore, in this study, we aimed to explore in vitro and in vivo anti-inflammatory activities of Mb-ME and understand its molecular action mechanism through the verification of target protein(s).

## 2. Results

### 2.1. Effect of Mb-ME on Nitric Oxide Production

**Figure 1:**
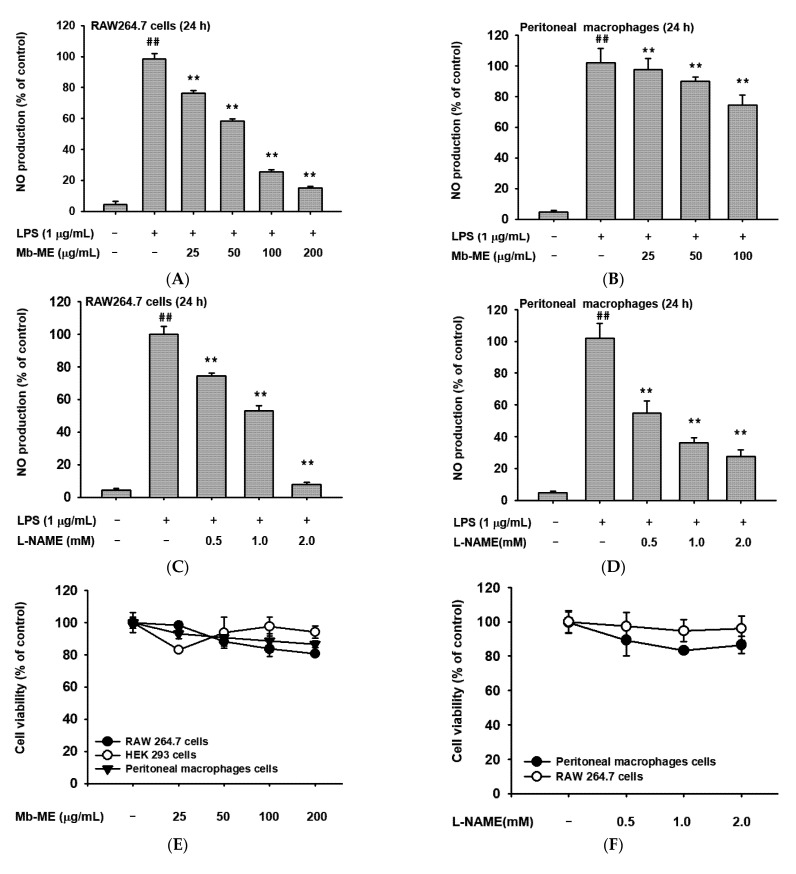

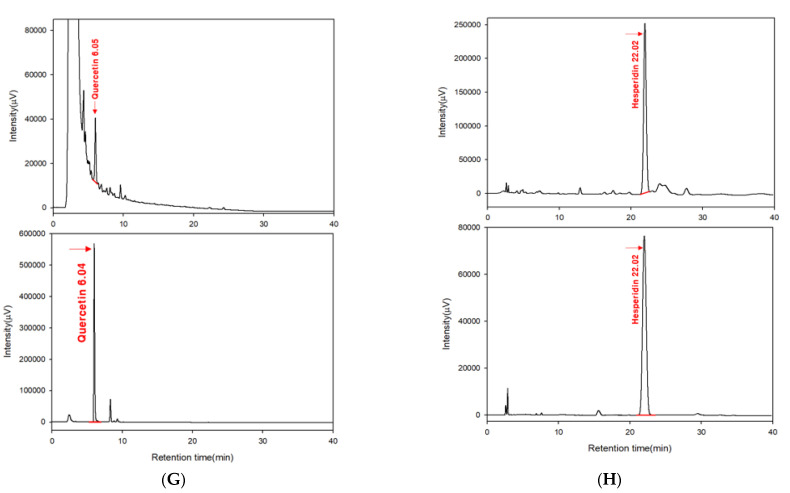
Effects of Mb-ME on the production of nitric oxide, cell viability, and HPLC. (**A**–**D**) Cells were pre-incubated with Mb-ME or L-NAME for 30 min and induced by LPS for 24 h. Nitric oxide production was determined. (**E**,**F**) The cytotoxicity of Mb-ME and L-NAME was investigated. (**G**,**H**) A phytochemical fingerprinting profile of Mb-ME was obtained by HPLC analysis. The lower panels in (**G**,**H**) are the HPLC profiles of standard compounds quercetin and hesperidin that appeared in 6.05 and 22.02 min, respectively. One millimole of L-NAME is 233.23 μg/mL. ^##^ *p* < 0.01 and ** *p* < 0.01 compared with the normal or control group.

Nitric oxide is a molecule that plays a crucial role in inflammation [21]. Therefore, we investigated nitric oxide production upon LPS stimulation. The production of nitric oxide was downregulated by Mb-ME in a dose-dependent manner in RAW264.7 cells and peritoneal macrophages (Figure 1A,B). Results also showed that the standard compound L-NAME inhibited in a dose-dependent manner the secretion of nitric oxide under the same conditions both in RAW264.7 cells and in peritoneal macrophages (Figure 1C,D) without cytotoxicity (Figure 1F). Similarly, Mb-ME showed no obvious cytotoxicity in three cell lines (RAW 264.7 cells, HEK293 cells, and peritoneal macrophage cells) (Figure 1E). Finally, HPLC analysis was performed to identify the flavonoids in Mb-ME. Quercetin and hesperidin (upper panels in Figure 1G,H), showing the same retention times (6.05 and 22.02 min, respectively) with their standard compounds (bottom panels in Figure 1G,H), were present in Mb-ME (Figure 1G,H). The content of hesperidin is 130.13 mg/g, while the content of quercetin is only 0.28 mg/g.

### 2.2. Effect of Mb-ME on the mRNA Expression of Inflammatory Genes

**Figure 2:**
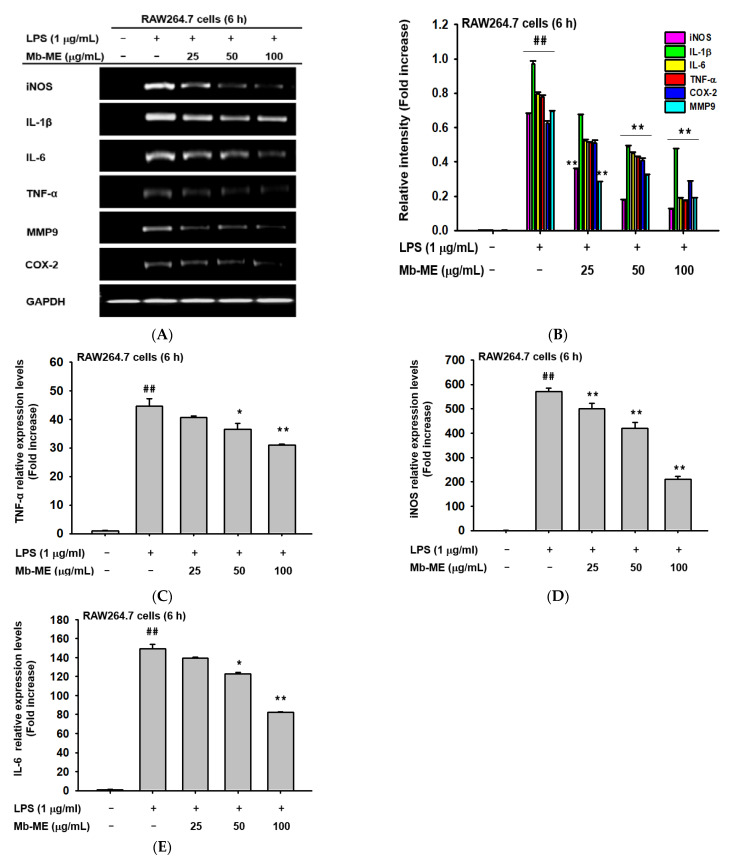
Inhibitory effects of Mb-ME on the expression of inflammatory genes. (**A**) RAW264.7 cells were pre-treated with Mb-ME for 30 min and incubated with LPS for 24 h. The expression levels of inflammatory genes were assessed by an RT-PCR. (**B**) The relative intensity of (**A**) was measured by ImageJ. (**C**–**E**) The expression levels of TNF-α, iNOS, and IL-6 were detected by a quantitative PCR. ^##^ *p* < 0.01 compared with the normal group; * *p* < 0.05 and ** *p* < 0.01 compared with the normal or control group.

To assess whether the decreasing trend of nitric oxide production was modulated by Mb-ME at the transcriptional level, the pro-inflammatory genes were investigated using PCR in LPS-induced conditions. In Figure 2A,B, semi-quantitative RT-PCR results showed that the expression of iNOS, IL-1β, IL-6, TNF-α, MMP9, and COX-2 was strongly stimulated by LPS treatment, while the expression was decreased in the presence of Mb-ME in a dose-dependent manner, especially at the concentration of 100 μg/mL. As expected, similar results were revealed by a quantitative RT-PCR. Mb-ME significantly blocked LPS-elevated expression levels of TNF-α, iNOS, and IL-6 (Figure 2C–E).

### 2.3. Effect of Mb-ME on the Translocation of Transcription Factors

**Figure 3:**
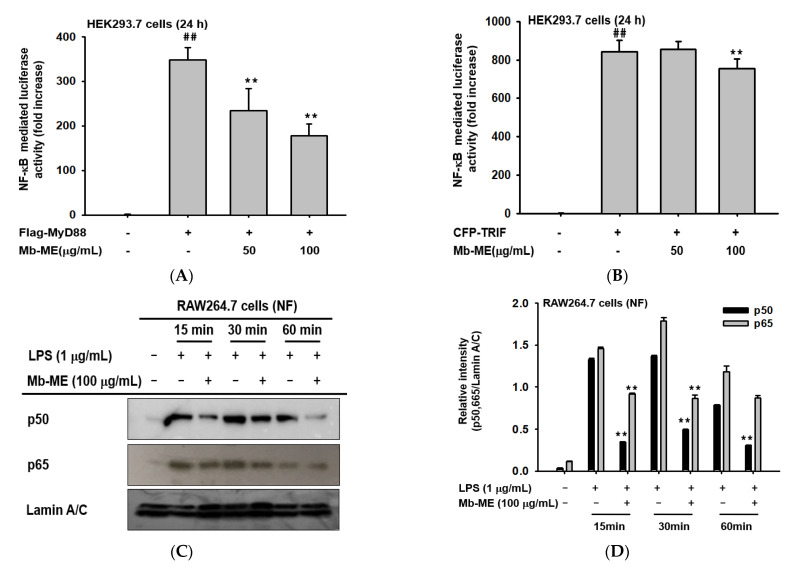
The anti-inflammatory effect of Mb-ME on the transcription level of the NF-κB signaling pathway. (**A**,**B**) HEK293 cells were transfected with NF-κB-Luc, MyD88, TRIF, and β-gal for 24 h before Mb-ME treatment. (**C**) The effects of Mb-ME on nuclear protein levels of p50, p65, and Lamin A/C were examined by immunoblotting. (**D**) The relative intensity of p50 and p65 (**C**) was measured by ImageJ. ^##^ *p* < 0.01 compared with the normal group and ** *p* < 0.01 compared with the control group.

Luciferase activities were measured using HEK293 cells to identify whether the inhibition of inflammation-related genes was regulated by Mb-ME through the NF-κB signaling pathway. NF-κB-mediated luciferase activity was dramatically triggered by MyD88 and inhibited by Mb-ME in a dose-dependent manner (Figure 3A). Meanwhile, Mb-ME significantly (*p* = 0.004589) altered TRIF-stimulated luciferase activity at 100 μg/mL but not at 50 μg/mL (Figure 3B), suggesting that the influence of Mb-ME on MyD88-mediated NF-κB signaling pathways is stronger than its influence on TRIF-mediated NF-κB activity. To further ascertain if Mb-ME did control NF-κB activation, the expression of p50 and p65, the most common heterodimers in the NF-κB signaling pathway, was evaluated at the protein level. Results showed that Mb-ME could largely attenuate both p50 and p65 at 15, 30, and 60 min, indicating that Mb-ME affected the NF-κB activating activity (Figure 3C,D).

### 2.4. Effect of Mb-ME in Activating the NF-κB Upstream Signaling Pathway

**Figure 4:**
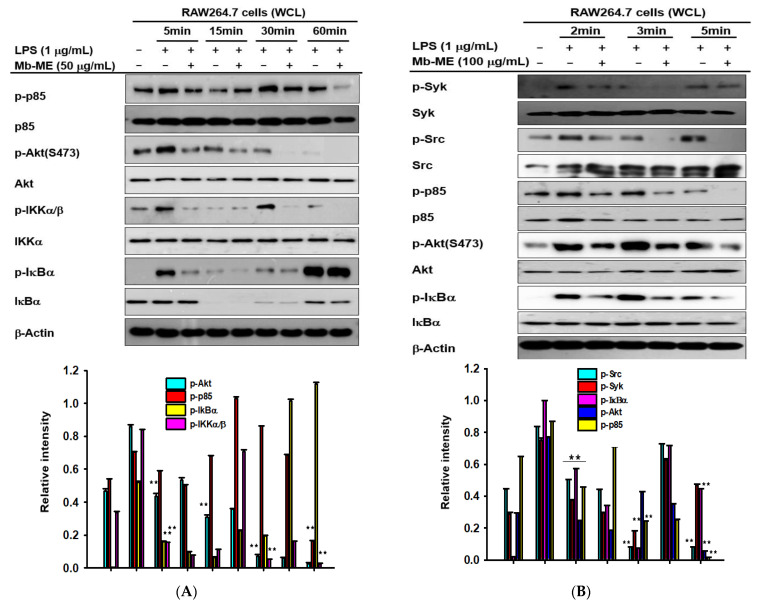

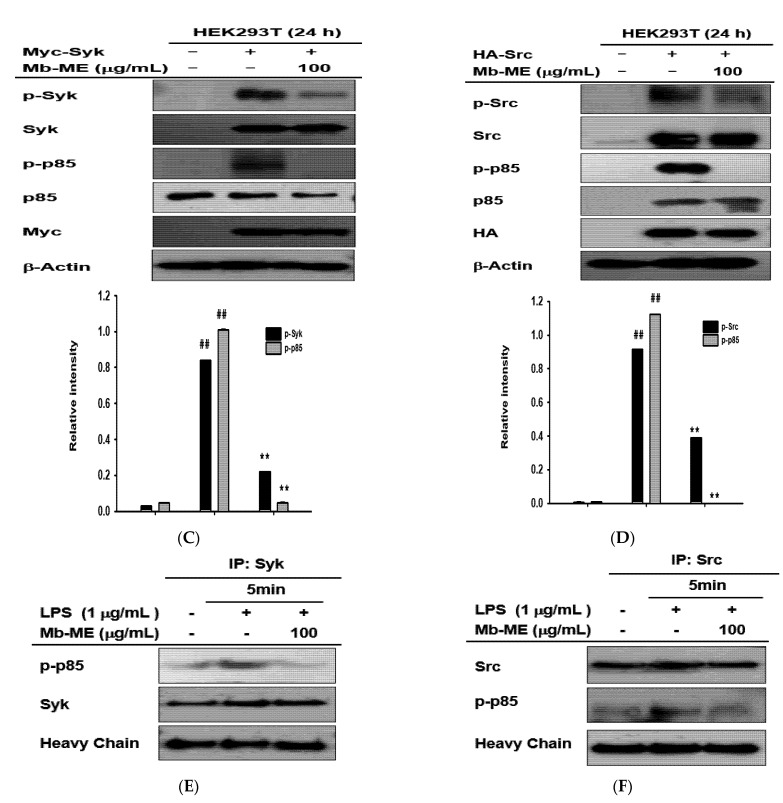
The regulatory effects of Mb-ME on NF-κB signaling. (**A**,**B**) RAW264.7 cells were pretreated with Mb-ME, stimulated by LPS for specific time points. The phosphorylated and total forms of interested proteins were measured by Western blot analysis. (**C**) HEK293 cells overexpressing HA-Src or Myc-Syk were treated with Mb-ME for 24 h. Phospho- and total forms of interested proteins were evaluated by immunoblotting. (**D**) RAW264.7 cells were induced with LPS in the presence or absence of Mb-ME for 5 min. (Lower panels in **A**–**D**) The relative intensity of signaling proteins (**A**,**B**) and Syk, Src or p85 (**C**,**D**) was measured by ImageJ. (**E**,**F**) The binding capacity of p-p85 to Src or Syk was examined by immunoprecipitation and immunoblotting analysis. ^##^ *p* < 0.01 compared to the normal group and ** *p* < 0.01 compared with the control.

To confirm whether Mb-ME also modulates the upstream molecules of the NF-κB signaling cascade, the expression of intracellular proteins in both total- and phosphor-forms was examined at different LPS-treatment time points in the presence or absence of Mb-ME. Phosphorylated Akt (S473) was diminished by Mb-ME after LPS induction compared with non-Mb-ME-treated groups at all time points (Figure 4A,B). Nevertheless, Mb-ME blocked the phosphorylation of IκBα only at 5 min. For the phosphorylation of IKKα/β, there was a clear inhibitory effect caused by Mb-ME except at 15 min. Since Mb-ME exerted inhibitory effects on Akt (S473) and IκBα induced by LPS at 5 min, some earlier time points were assessed to further evaluate the upstream molecules. The blocking trend appeared after Mb-ME treatment at almost all time points, especially the phosphorylation of IκBα and p85 (Figure 4B). Syk and Src are essential kinases for the initiation of signaling and could be activated to regulate various activities. Therefore, the phosphorylation of Syk and Src was investigated. As Figure 4B shows, Mb-ME offered a significant inhibitory effect on phosphorylation of Src at all time points, while the pattern of Syk could be observed only at 2 and 3 min after treating with LPS. To further determine the putative target protein of Mb-ME, an overexpression assay of Syk and Src was conducted. In Figure 4C,D, both Syk and Src overexpression increased the levels of phosphorylated forms of p85, Syk, and Src, whereas these events were significantly suppressed by Mb-ME treatment. To verify the inhibitory effect of Mb-ME on Src and Syk, immunoprecipitation was performed to evaluate the binding activity between Src and Syk and their substrates. As seen in Figure 4E,F, complex formation of p85 with either Src or Syk was abolished by Mb-ME, strongly implying Src and Syk as direct targets of Mb-ME.

### 2.5. Effect of Mb-ME on In Vivo EtOH/HCl-Induced Gastritis

**Figure 5:**
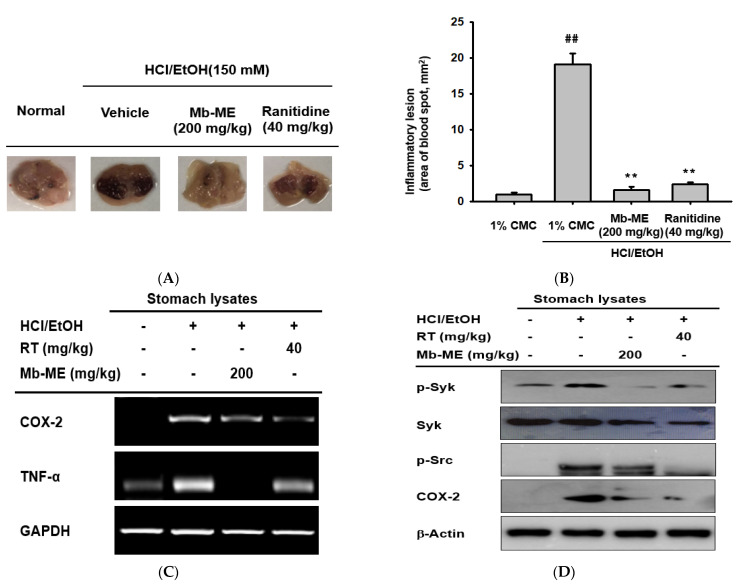
In vivo effect of Mb-ME on HCl/EtOH-induced gastritis. (**A**,**B**) Mice were orally injected with Mb-ME or ranitidine and gastritis was induced by HCl/EtOH. The mucosal erosive areas were calculated with ImageJ. (**C**,**D**) The mRNA and protein were isolated from stomach tissues. mRNA expression of COX-2 and TNF-α and protein levels of COX-2, p-Syk, Syk, and p-Src in gastritis stomach samples were evaluated by a PCR and immunoblotting, respectively. ^##^ *p* < 0.01 compared to the normal group and ** *p* < 0.01 compared to the control.

To assess the therapeutic function of Mb-ME in inflammatory diseases in vivo, we used a gastritis mouse model triggered by HCl/EtOH. Ranitidine, which is used to treat and prevent ulcers in the stomach and intestines, was used as a positive control. Based on our previous study [22,23,24,25] and in vitro activity of Mb-ME showing a concentration range from 0 to 100 μg/mL, we decided doses of Mb-ME and ranitidine up to 200 and 40 mg/kg, respectively. As shown in Figure 5A,B, Mb-ME strongly ameliorated the inflammatory lesion to a degree similar to that of ranitidine. The expression of COX-2 and TNF-α in stomach tissues was measured to confirm whether in vivo results were similar with in vitro data. As expected, the expression of both COX-2 and TNF-α exhibited a decreasing trend on Mb-ME treatment (Figure 5C). Moreover, the Western blotting results implied that Mb-ME strongly diminishes the enhanced COX-2 level triggered by HCl/EtOH in stomach lysates and the anti-gastritis ability of Mb-ME was almost the same as that of ranitidine (Figure 5D). To confirm whether Mb-ME could also modulate the NF-κB in vivo condition, the expression of p-Syk and p-Src in stomach tissues was assessed. As expected, Mb-ME decreased the levels of both, especially the phosphorylated form of Syk (Figure 5D).

## 3. Discussion

*Malus baccata* (L.) Borkh. is an important traditional medical plant. Several studies have revealed its anti-obesity, anti-oxidant, and immunomodulation activities, emphasizing its pharmacological importance [19]. However, the molecular mechanisms of this plant, including those of its leaves and shoots, in inflammatory responses remain poorly understood. The goal of this study was to reveal the molecular mechanism of Mb-ME-mediated anti-inflammatory activity using LPS-stimulated macrophages. Therefore, we investigated the anti-inflammatory effect of Mb-ME using RAW264.7 cells, as well as an acute gastritis murine model. Moreover, the target molecules of Mb-ME were explored in the NF-κB signaling pathways.

Nitric oxide is recognized as a mediator and regulator molecule that plays several roles in immunity and inflammation [26]. First, the production of nitric oxide was inhibited by Mb-ME without significant cytotoxicity upon LPS-induced RAW264.7 cells and primary peritoneal macrophages (Figure 1A–F). As shown in Figure 1G,H, Mb-ME contained quercetin and hesperidin, flavonoids famous for their anti-inflammatory and anti-oxidant effects. The effect of Mb-ME on Src and Syk phosphorylation and nitric oxide production might be attributed to these compounds. Further evidence is required to support this.

Inducible nitric oxide synthase (iNOS) is an enzyme that synthesizes nitric oxide [27]. Thus, the alteration of Mb-ME on mRNA expression of iNOS was investigated. As we expected, semi-quantitative and quantitative PCR results suggest that mRNA expression of iNOS is blocked by Mb-ME in a dose-dependent manner. Mb-ME also suppressed other inflammatory genes, such as IL-6, MMP9, TNF-α, IL-1β, and COX-2 (Figure 2). In both innate and adaptive immune cells, NF-κB is a central mediator that mediates cell survival, differentiation, and activation of pro-inflammatory genes expression such as cytokines, chemokines, and coagulation factors [28]. Our results indicate that the nuclear translocation of p50 and p65, two subunits of NF-κB, was suppressed by Mb-ME. Moreover, Mb-ME simultaneously inhibited the luciferase activity of NF-κB that was triggered by MyD88 and TRIF (Figure 3). Our experiment implies that Mb-ME plays a suppressive role in nitric oxide production and pro-inflammatory genes, accomplished by blocking the nuclear transcription and activity of NF-κB.

The nuclear transcription of NF-κB involves the activation of IκBα (inhibitors of NF-κB), Akt, and IκB kinase (IKK) [29,30]. Moreover, phosphatidyl-inositol-3-kinase (PI3K), Syk, and Src are upstream molecules of the NF-κB signaling pathway. Mb-ME suppressed the phosphorylation of Akt, IKKα/β, and IκBα in LPS-stimulated macrophages at most of the tested time points (Figure 4A). A significant reduction in IκBα at 5 min was observed. In certain cytokine responses, it is believed that Src and Syk family kinases usually act downstream of the Toll-like receptors by interacting with Src and Syk at earlier time points. In agreement with our expectation, the phosphorylation of Src, Syk, and p85 was enhanced by LPS stimulation while it was downregulated by Mb-ME treatment at 2, 3, and 5 min (Figure 4B). Our hypothesis is that Mb-ME targeted Src and Syk. To verify this, overexpression of Src and Syk was employed. Consistent with Figure 4B, the phosphorylation of Src and Syk and their downstream enzyme, p85, was inhibited by Mb-ME, which implied Src and Syk as direct targets during Mb-ME-mediated anti-inflammatory activities (Figure 4C,D). Molecular complex formation between Src/Syk and PI3K was blocked by Mb-ME (Figure 4E,F). Moreover, an in vivo experiment confirmed that the oral administration of Mb-ME ameliorated HCl/EtOH-induced acute gastritis (Figure 5). In general, the anti-inflammatory activity of Mb-ME is driven by the suppression of p-Syk and p-Src in NF-κB pathways.

## 4. Materials and Methods

### 4.1. Materials

The methanol extract of *Malus baccata* (L.) Borkh (Mb-ME) was obtained from the Plant Extract Bank in the Plant Diversity Research Center (Daejeon, Korea). The RAW264.7 (murine macrophages) cells and HEK293 (human embryonic kidney) cells were purchased from ATCC (Rockville, MD, USA). The reagents used for culturing cells were purchased from Gibco (Grand Island, NY, USA). Lipopolysaccharide (LPS), polyethylenimine (PEI), ranitidine, sodium carboxymethyl cellulose (Na-CMC), and other chemicals were obtained from Sigma Chemical Co. (St. Louis, MO, USA). Phospho-specific and total antibodies recognizing β-actin, IκBα, p50, IKKα/β, HA, Src, p65, Syk, p85, Lamin A/C, Akt, p85, IκBα, and Myc were purchased from Cell Signaling (Beverly, MA, USA).

### 4.2. Plant Information and Extraction Methods

*Malus baccata* (L.) Borkh. was collected from Xiao Longman National Forest Park, Mentougou district, Beijing, China, and identified by Dr. Zhiyun Zhang at the Institute of Botany in May 2012. A voucher specimen (accession number KRIB 0041120) of the retained material is preserved at the herbarium of KRIBB. The leaves and shoots of *Malus baccata* (19 g) were extracted with 1 L of 99.9% (v/v) methanol under repeated sonication (15 min) and rest (2 h) for 3 days at 45 °C. The resultant product was filtered with non-fluorescence cottons and concentrated by a rotary evaporator (N-1000SWD, EYELA) under reduced pressure at 45 °C. Finally, a total 4.09 g of methanol extract of *Malus baccata* was obtained by freeze-drying.

### 4.3. Cell Culture

RAW264.7 cells and HEK293 cells were cultured in RPMI1640 supplemented with 10% fetal bovine serum or DMEM supplemented with 5% fetal bovine serum, respectively. Cells were incubated in 5% CO_2_ at 37 °C and passaged two times a week.

### 4.4. Drug Preparation

A stock solution (100 mg/mL) of Mb-ME solved with 100% DMSO was first diluted with DMSO to have various concentrations of Mb-ME (25, 50, and 100 mg/mL). For preparing in vitro working concentrations (25, 50, and 100 μg/mL) of Mb-ME, a culture medium was used for the final dilution. Mb-ME was prepared in 1% Na-CMC for the in vivo experiment [31]. LPS (stock solution 1 mg/mL) was also diluted with the culture medium to reach a working concentration (1 μg/mL).

### 4.5. Animals

ICR mice and C57BL/6 mice were obtained from Dae Han Bio Link (Osong, Korea) [32]. Food pellets and water were supplied ad libitum [33]. All studies were performed in accordance with guidelines established by the Sungkyunkwan University Institutional Animal Care and Use Committee.

### 4.6. Preparation of Peritoneal Macrophages

To obtain peritoneal macrophages, sterile thioglycollate broth was intraperitoneally injected into male C57BL/6 mice and lavaged for 4 days [34]. The exudates were washed with an RPMI medium containing 10% FBS [35]. Peritoneal macrophages were plated in 100 mm tissue culture dishes.

### 4.7. Nitric Oxide Production Assay

RAW264.7 cells and peritoneal macrophages were seeded into 96-well plates. The cells were pre-treated with Mb-ME for 30 min and induced with LPS for 24 h, and 100 μL of the supernatant was transferred to a new 96-well plate and mixed with Griess reagent. The nitric oxide production level was calculated by measuring absorbance at 540 nm [36].

### 4.8. Cell Viability Assay

The cells were treated with the indicated concentration of Mb-ME or L-NAME for 24 h [37]. Then, 10 μL of MTT solution was distributed to each well. The reaction was terminated by adding 100 μL of MTT stopping solution. The cytotoxicity of Mb-ME and L-NAME was explored by determining the absorbance at 570 nm.

### 4.9. High-Performance Liquid Chromatography (HPLC)

HPLC was performed with the Jasco HPLC system including a UV-Vis detector to identify the bioactive components of Mb-ME [15,38]. The injection volume was 10 μL, and the flow rate was 1.0 μL/min. Quercetin and hesperidin were employed as the standard compounds [39]. The HPLC conditions for analyzing quercetin and hesperidin in Mb-ME are explained in Table 1.

### 4.10. Plasmid Transfection and Luciferase Reporter Assay

HEK293 cells were transfected with Syk, Src, MyD88, TRIF, or NF-κB Luc via PEI for 24 h [40]. The above cells were further treated with Mb-ME for 24 h [41]. A luciferase assay was performed to test the effect of Mb-ME on NF-κB activity. Cells overexpressing Syk or Src were further incubated with Mb-ME for 24 h, harvested, and subjected to immunoblotting.

### 4.11. mRNA Analysis by a Reverse Transcription Polymerase Chain Reaction

Following pretreatment by Mb-ME for 30 min, RAW264.7 cells were induced with LPS for 6 h. Total RNA was isolated using TRIzol reagent. Complementary DNA was synthesized using mRNA as the template. A reverse transcription polymerase chain reaction (RT-PCR) and a real-time PCR were performed [42]. All the primers are listed in Table 2 and Table 3.

### 4.12. Preparation of Total Cell and Nuclear Lysates

Experimental RAW264.7 cells and HEK293 cells were harvested and resuspended in lysis buffer. For nuclear fractionation, RAW264.7 cells were harvested and resuspended in nuclear protein extraction buffer [43]. The nuclear extract and whole protein lysates were isolated by centrifugation and stored at −80 °C.

### 4.13. Immunoblotting

A Bradford assay was employed to determine the protein concentration of samples. The proteins of whole cells or nuclear lysates were then subjected to immunoblotting, as performed previously [44]. The phosphorylated or total proteins of p85, Akt, IKKα/β, Syk, Src, Myc, HA, COX-2, and β-actin were visualized using an ECL system [45].

### 4.14. Immunoprecipitation

Proteins was isolated from RAW264.7 cells treated with Mb-ME and LPS. Lysates containing equal amounts of proteins were incubated with 3 μL Syk- or Src-specific antibodies at 4 °C overnight. The protein complexes were mixed with 10 μL of protein A-conjugated agarose beads for 4 h at 4 °C [46]. Protein complexes were washed, and the beads were boiled. The proteins were isolated and then subjected to immunoblotting.

### 4.15. HCl/EtOH-Induced Gastritis

The acute gastritis model was induced with EtOH/HCl in 25 ICR mice. Fasted mice (6 mice/group) were orally administered Mb-ME or ranitidine twice per day for 2 days [31]. One hour after the final oral administration, the mice were orally injected with 400 μL of 60% ethanol in 150 mM HCl [47]. The mice were anesthetized and sacrificed after 1 h. The mRNA and protein were isolated from stomach tissue lysates for an RT-PCR and immunoblotting analysis, respectively.

### 4.16. Statistical Analysis

Data are expressed as the mean ± the standard deviation of experiments performed with triplicate samples for the in vitro experiments or with septuplicate samples for the in vivo experiments. SPSS (Ver. 22) was employed to evaluate the differences between groups. *p* < 0.05 was considered statistically significant.

## 5. Conclusions

**Figure 6:**
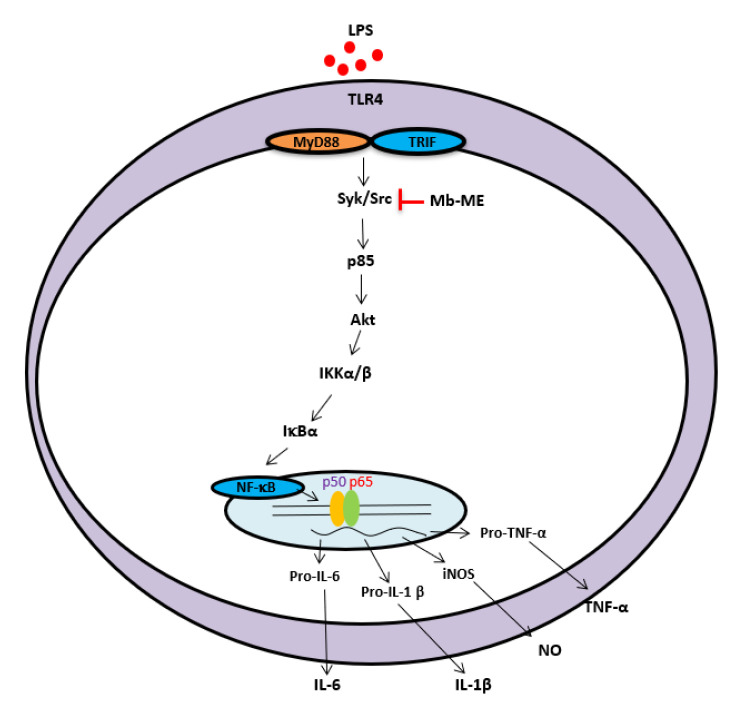
The potential inhibitory pathway of Mb-ME-mediated anti-inflammatory activities.

In summary, in this study, Mb-ME reduced the production of nitric oxide; decreased the mRNA expression of pro-inflammatory genes such as TNF-α, IL-6, and iNOS; and relieved acute gastritis symptoms triggered by HCl/EtOH treatment. This extract also showed the suppression of Src and Syk activities, leading to a reduction of the NF-κB activation pathway under LPS-activated conditions, as summarized in Figure 6. Our results imply that Mb-ME provides an anti-inflammatory effect by targeting the NF-κB signaling pathway both in vivo and in vitro. The potential anti-inflammatory function suggests Mb-ME as an ideal therapeutic candidate in inflammatory illness. Therefore, we will continue a more detailed pre-clinical study against various gastritis diseases with the ethanol or water extracts of this plant.

## Figures and Tables

**Table 1:** HPLC conditions.

Analyzing Compound	Quercetin	Hesperidin
Column	CAPCELL PAK C18 MG, 4.6 mm I.D. × 250 mm	CAPCELL PAK C18 MG, 4.6 mm I.D. × 250 mm
Wavelength (nm)	254	285
Mobile phase	A: 2% acetic acid in water B: 0.5% acetic acid in water: CAN = 50:50	Methanol: water of acetic acid (pH 3) = 30:70
Gradient or isocratic conditions	0: A (28%), B (72%) 20 min: B (100%) 30 min: B (100%)	40 min: 100%
Oven temperature (°C)	35	50

**Table 2:** Sequences of mouse primers used in the semi-quantitative RT-PCR.

Name	Primer	Sequence (5′ to 3′)
iNOS	Forward	GTGAAGAAAACCCCTTGTGCTG
	Reverse	AGTTCCGAGCGTCAAAGACC
IL-1β	Forward	CAGGATGAGGACATGAGCACC
	Reverse	CTCTGCAGACTCAAACTCCAC
IL-6	Forward	GCCTTCTTGGGACTGATGCT
	Reverse	TGGAAATTGGGGTAGGAAGGAC
TNF-α	Forward	TTGACCTCAGCCGTGAGTTG
	Reverse	CCTGTAGCCCACGTCGTAGC
MMP-9	Forward	TCTTCCCCAAAGACCTGAAA
	Reverse	TGATGTTATGATGGTCCCAC
COX-2	Forward	CACTACATCCTGACCCACTT
	Reverse	ATGCTCCTGCTTGAGTATGT
GADPH	Forward	ACCACAGTCCATGCCATCAC
	Reverse	CCACCACCCTGTTGCTGTAG

**Table 3:** Sequences of mouse primers used in the real-time PCR.

Name	Primer	Sequence (5′ to 3′)
TNF-α	Forward	TGCCTATGTCTCAGCCTCTT
	Reverse	GAGGCCATTTGGGAACTTCT
iNOS	Forward	CGAAACGCTTCACTTCCAA
	Reverse	TGAGCCTATATTGCTGTGGCT
IL-6	Forward	GTCCTTCCTACCCCAATTTCCA
	Reverse	TAACGCACTAGGTTTGCCGA
GADPH	Forward	GGGTCCCAGCTTAGGTTCATC
	Reverse	TACGGCCAAATCCGTTCACA

## Data Availability

The data are contained within the article.

## Abbreviations

AP-1	Activator protein 1
COX-2	Cyclooxygenease-2
HPLC	High-performance liquid chromatography
iNOS	Inducible nitric oxide synthase
IL-1β	Interleukin 1β
IL-6	Interleukin 6
JAK-STAT	Janus kinase-signal transducer and activator of transcription
LPS	Lipopolysaccharides
Mb-ME	*Malus baccata* methanol extract
MyD88	Myeloid differentiation primary response 88
MMP9	Matrix metallopeptidase 9
NF-κB	Nuclear factor-κB
Na-CMC	Sodium carboxymethyl cellulose
PRR	Pattern recognition receptor
pp85	Phospho-PI3 kinase p85
RT-PCR	Reverse transcription polymerase chain reaction
Src	Non-receptor tyrosine kinase
Syk	Spleen tyrosine kinase
TLR	Toll-like receptors
TRIF	TIR-domain-containing adapter-inducing interferon-β
TIRAP	TIR domain containing adaptor protein
TRAM	TRIF-related adaptor molecule
TNF-α	Tumor necrosis factor α
TAK1	Transforming growth factor beta-kinase 1
